# Eggshell membrane-based biomaterials for tissue regeneration: a systematic review of preclinical evidence

**DOI:** 10.3389/fbioe.2026.1815447

**Published:** 2026-06-25

**Authors:** Vivekanand Kattimani, Shahabe Saquib Abullais, Salma Abubaker Abbas Ali, Mohasin Abdul Khader, Shaik Mohammed Asif, Ratna Kumari Anantha, Rahul Tiwari

**Affiliations:** 1 Department of Oral and Maxillofacial Surgery, Narsinhbhai Patel Dental College & Hospital, Sankalchand Patel University, Visnagar, Gujarat, India; 2 Department of Clinical Research, SIBAR Institute of Dental Sciences, Guntur, Andhra Pradesh, India; 3 Department of Periodontics and Community Dental Science, College of Dentistry, King Khalid University, Abha, Saudi Arabia; 4 Department of Oral Diagnosis, Oral Biology and Periodontology, King Khalid University, Abha, Saudi Arabia; 5 Department of Periodontics, College of Dentistry, King Khalid University, Abha, Saudi Arabia; 6 Department of Oral Diagnosis, Oral Biology and Periodontology, College of Dentistry, King Khalid University, Abha, Saudi Arabia; 7 Department of Dental Research Cell, Dr. D. Y. Patil Dental College and Hospital, Dr. D. Y. Patil Vidyapeeth (Deemed to be University), Pune, India

**Keywords:** biomaterials, eggshell membrane, preclinical studies, regenerative medicine, tissue regeneration

## Abstract

**Background:**

Limitations of conventional wound dressings have spurred the exploration of regenerative biomaterials capable of actively promoting tissue repair. Eggshell membrane (ESM) is emerging as a low-cost, biocompatible scaffold with significant potential in advanced wound care applications.

**Aim:**

To evaluate the therapeutic potential of ESM-based wound dressings in enhancing the wound-healing process.

**Methods:**

A systematic review was conducted following the Preferred Reporting Items for Systematic Reviews and Meta-Analyses (PRISMA) guidelines. Comprehensive searches were performed across PubMed, Scopus, ScienceDirect, and ProQuest, covering all records up to 22 June 2024. Only English-language studies investigating eggshell membrane as a wound care biomaterial were included. Study selection adhered to predefined eligibility and inclusion criteria.

**Results:**

From 599 identified records, 36 studies met the inclusion criteria. Fifteen studies involved *in vivo* experiments (eight in rats, three in rabbits, three in mice, and one in murine models), with ten also incorporating *in vitro* analyses. Eight studies were exclusively *in vitro*. Overall, 15 studies used *in vivo* methods and 18 utilized *in vitro* approaches. ESM-based wound dressings consistently demonstrated promising outcomes, accelerating wound repair and tissue regeneration, and often outperforming untreated controls or conventional gauze dressings. A meta-analysis was not feasible due to heterogeneity in study designs, experimental models, and outcome measures.

**Conclusion:**

This review highlights the considerable potential of eggshell membrane-based dressings as regenerative biomaterials for the management of complex wounds. While ESM shows strong promise as a scaffold for wound healing, further standardized and clinically focused research is necessary to establish robust evidence supporting its translational and therapeutic applications.

**Systematic Review Registration:**

https://www.crd.york.ac.uk/PROSPERO/view/CRD420260551038, identifier CRD420260551038.

## 1 Introduction

A wound occurs when the integrity of the skin, mucous membrane or internal organs is disrupted. Effective cleansing and appropriate dressing are critical to prevent infection and associated complications (Kujath and Michelsen, 2008; Wilkins and Unverdorben, 2016). Globally, an estimated 312.9 million individuals experience surgically induced wounds each year, while nearly 76 million people suffer from wounds associated with comorbid conditions such as diabetes, obesity, and cardiovascular disease (Weiser et al., 2016). Improper management of skin injuries can often lead to long-term disability and, in severe cases, mortality.

In recent years, eggshell membranes (ESM) have attracted considerable research attention due to their wide availability, biocompatibility, biodegradability, and inherent antimicrobial and anti-inflammatory properties, which are critical for efficient wound healing (Park et al., 2016; Kulshreshtha et al., 2020). Growing interest in natural biomaterials has prompted researchers to investigate ESM as a wound care substrate using *in vitro* cell line studies and *in vivo* animal models. Consequently, a review is proposed to systematically evaluate existing research and assess the potential of ESM as a wound care material.

Eggshells and ESM constitute approximately 10%–12% and 1.02% of the total egg structure, respectively (Zhou et al., 2008), generating an estimated 883,800 tons of ESM and 9.531 million tons of eggshell by-products annually. ESM is a predominantly protein-based biomaterial, composed of approximately 90% protein, 3% lipids, and 2% carbohydrates, with trace amounts of calcium and magnesium (Han et al., 2023). Proteomic and bioinformatic investigations have identified more than 500 proteins within ESM, including collagens, glycoproteins, avian β-defensins, and lysozyme, along with biologically significant carbohydrates such as hyaluronic acid (Kulshreshtha et al., 2022). These constituents impart antioxidant, anti-inflammatory, antimicrobial, and regenerative properties that are highly beneficial for wound healing applications.

Although previous reviews have explored ESM in relation to bone health, osteoarthritis, energy systems, adsorbents, and pharmaceutical applications (Elkhenany et al., 2022; Baláž et al., 2021; Fladerer and Grollitsch, 2024; Rosaiah et al., 2024; García-Muñoz et al., 2024; Liang et al., 2024; Torres-Mansilla et al., 2023; Mensah et al., 2023a; Gavali et al., 2024; Mittal et al., 2016), a comprehensive analysis focused specifically on wound care is lacking. This review, therefore, aims to evaluate the potential of ESM as a wound healing material by critically assessing its performance across different material formats—including native membranes, powders, hydrogels, composites, and bioengineered dressings using evidence from *in vitro* studies and, animal models. Furthermore, it highlights key translational challenges such as processing techniques, bioavailability, standardization, and clinical readiness that must be addressed to facilitate the transition of ESM from laboratory research to practical clinical wound care applications.

## 2 Materials and methods

A systematic literature search was conducted using the keywords eggshell membrane, wound care, and wound healing, combined with Boolean operators and applied to the title, abstract, and keyword fields of the electronic databases PubMed, Scopus, ProQuest, and ScienceDirect (Table 1). English-language publications indexed in these databases from inception to 22 June 2024 were included. Manual screening of reference lists was not performed; only records identified through the predefined database search and grey literature sources were considered. Any discrepancies between reviewers were resolved through consensus-based discussion. Consistent with Preferred Reporting Items for Systematic Reviews and Meta-Analyses (PRISMA) guidelines, a formal risk-of-bias assessment was conducted, as the objective of this review was to map the available evidence rather than to assess the effectiveness of interventions (Tricco et al., 2018). Eggshell membrane as a wound care biomaterial–a systematic review was registered at Prospero (Prospero ID: CRD420260551038).

**TABLE 1:** Search strategy.

Search engine	Search string	Search dates	Number of search results
Pubmed	(“wound healing”[MeSH Terms] OR (“wound”[All Fields] AND “healing”[All Fields]) OR “wound healing”[All Fields] OR ((“injuries”[MeSH Subheading] OR “injuries”[All Fields] OR “wounds”[All Fields] OR “wounds and injuries”[MeSH Terms] OR (“wounds”[All Fields] AND “injuries”[All Fields]) OR “wounds and injuries”[All Fields] OR “wound s”[All Fields] OR “wounded”[All Fields] OR “wounding”[All Fields] OR “woundings”[All Fields] OR “wound”[All Fields]) AND “care”[All Fields]) OR ((“injuries”[MeSH Subheading] OR “injuries”[All Fields] OR “wounds”[All Fields] OR “wounds and injuries”[MeSH Terms] OR (“wounds”[All Fields] AND “injuries”[All Fields]) OR “wounds and injuries”[All Fields] OR “wound s”[All Fields] OR “wounded”[All Fields] OR “wounding”[All Fields] OR “woundings”[All Fields] OR “wound”[All Fields]) AND (“therapeutics”[MeSH Terms] OR “therapeutics”[All Fields] OR “treatments”[All Fields] OR “therapy”[MeSH Subheading] OR “therapy”[All Fields] OR “treatment”[All Fields] OR “treatment s”[All Fields])) OR ((“injuries”[MeSH Subheading] OR “injuries”[All Fields] OR “wounds”[All Fields] OR “wounds and injuries”[MeSH Terms] OR (“wounds”[All Fields] AND “injuries”[All Fields]) OR “wounds and injuries”[All Fields] OR “wound s”[All Fields] OR “wounded”[All Fields] OR “wounding”[All Fields] OR “woundings”[All Fields] OR “wound”[All Fields]) AND (“bandages”[MeSH Terms] OR “bandages”[All Fields] OR “dressing”[All Fields] OR “dressings”[All Fields] OR “dress”[All Fields] OR “dressed”[All Fields] OR “dresses”[All Fields] OR “dressing s”[All Fields])) OR ((“therapeutics”[MeSH Terms] OR “therapeutics”[All Fields] OR “treatments”[All Fields] OR “therapy”[MeSH Subheading] OR “therapy”[All Fields] OR “treatment”[All Fields] OR “treatment s”[All Fields]) AND (“injuries”[MeSH Subheading] OR “injuries”[All Fields] OR “wounds”[All Fields] OR “wounds and injuries”[MeSH Terms] OR (“wounds”[All Fields] AND “injuries”[All Fields]) OR “wounds and injuries”[All Fields] OR “wound s”[All Fields] OR “wounded”[All Fields] OR “wounding”[All Fields] OR “woundings”[All Fields] OR “wound”[All Fields]))) AND (((“egg shell”[MeSH Terms] OR (“egg”[All Fields] AND “shell”[All Fields]) OR “egg shell”[All Fields] OR “eggshell”[All Fields] OR “eggshells”[All Fields]) AND (“membranal”[All Fields] OR “membrane s”[All Fields] OR “membraneous”[All Fields] OR “membranes”[MeSH Terms] OR “membranes”[All Fields] OR “membrane”[All Fields] OR “membranous”[All Fields])) OR ((“egg shell”[MeSH Terms] OR (“egg”[All Fields] AND “shell”[All Fields]) OR “egg shell”[All Fields] OR “eggshell”[All Fields] OR “eggshells”[All Fields]) AND (“membranal”[All Fields] OR “membrane s”[All Fields] OR “membraneous”[All Fields] OR “membranes”[MeSH Terms] OR “membranes”[All Fields] OR “membrane”[All Fields] OR “membranous”[All Fields])) OR ((“egg shell”[MeSH Terms] OR (“egg”[All Fields] AND “shell”[All Fields]) OR “egg shell”[All Fields] OR “eggshell”[All Fields] OR “eggshells”[All Fields]) AND (“layer”[All Fields] OR “layer s”[All Fields] OR “layered”[All Fields] OR “layering”[All Fields] OR “layerings”[All Fields] OR “layers”[All Fields])) OR ((“egg shell”[MeSH Terms] OR (“egg”[All Fields] AND “shell”[All Fields]) OR “egg shell”[All Fields] OR “eggshell”[All Fields] OR “eggshells”[All Fields]) AND (“membranal”[All Fields] OR “membrane s”[All Fields] OR “membraneous”[All Fields] OR “membranes”[MeSH Terms] OR “membranes”[All Fields] OR “membrane”[All Fields] OR “membranous”[All Fields])))	Time of inception (1984) till 22 June 2024	58 search results without any filters
Scopus	(TITLE-ABS-KEY (eggshell AND membrane) OR TITLE-ABS-KEY (eggshell AND layer) OR TITLE-ABS-KEY (eggshell AND membranous)) AND (TITLE-ABS-KEY (wound AND care) OR TITLE-ABS-KEY (wound AND dressing) OR TITLE-ABS-KEY (wound AND treatment) OR TITLE-ABS-KEY (treatment AND of AND wounds) AND TITLE-ABS-KEY (wound AND healing))	Time of inception till 22 June 2024	34 search results
Proquest	(eggshell membrane) AND (Wound care) AND (Wound heal)	Time of inception till 22 June 2024	104 search results
Science direct	(eggshell membrane) AND (Wound care) AND (Wound heal)	Time of inception till 22 June 2024	403 search results

### 2.1 Method of selection

All bibliographic records were imported into and managed using Zotero software. After duplicate records were removed, two independent teams, each consisting of three reviewers, screened the articles. The relevance of each study was assessed according to predefined inclusion and exclusion criteria.

#### 2.1.1 Inclusion criteria

*In vivo* and *In vitro* studies evaluating ESM as a wound care material.

#### 2.1.2 Exclusion criteria

Case reports, case series, books, editorials, letters, conference proceedings, other non–peer-reviewed publications, and review articles.

The PRISMA extension for reviews was used to present the study selection process through a flow diagram. All included articles were comprehensively summarized and systematically charted. Extracted data included author information, material composition, study type (*in vivo*, *in vitro*, or both), and the datasets used.

## 3 Results

**FIGURE 1:**
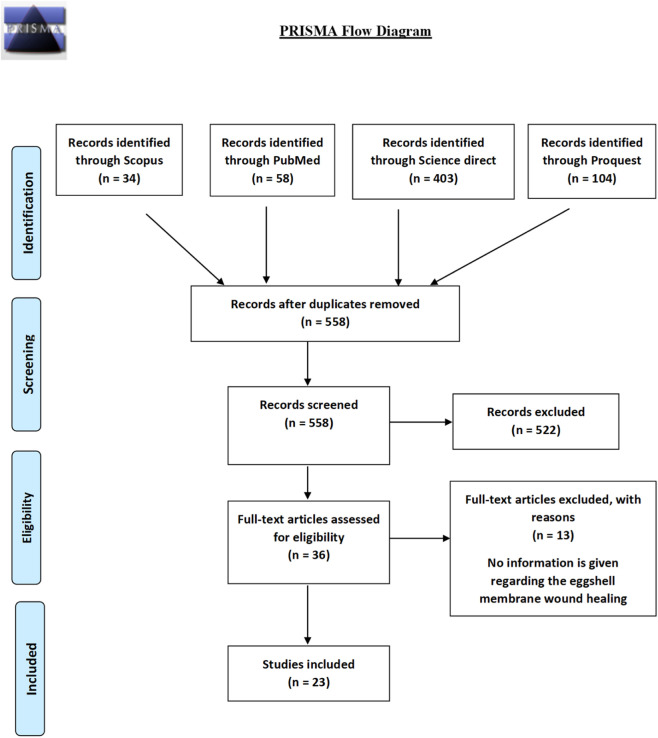
PRISMA flow diagram.

A systematic search of PubMed (58 records), Scopus (34), ScienceDirect (403), and ProQuest (104) identified 599 articles after removal of 41 duplicates. Following initial title and abstract screening, 522 records were excluded, leaving 36 full-text articles for eligibility assessment (Figure 1). Of these, 13 articles were excluded after full-text review for not meeting the inclusion criteria (Supplementary Table S1). Ultimately, 23 sources of evidence were included in the final review. A meta-analysis was not feasible due to heterogeneity in study designs, experimental models, and outcome measures.

### 3.1 Characteristics of the included studies

All twenty-three included studies were preclinical laboratory investigations conducted using *in vivo* and *in vitro* models (Tables 2, 3). Of these, eight studies were performed in rats, three in rabbits, three in mice, one involved a murine model and the remaining eight were exclusively *in vitro* studies.

**TABLE 2:** Characteristics of *in vivo* studies of ESM as a wound care material.

Author (Year)	Animal model	Wound type	ESM material/Format	Comparison groups	Key outcomes	Percentage of wound healing and duration of full closure
Guarderas et al. (2016)	Sprague-Dawley rat	7 mm full-thickness skin wound	Natural ESM membrane	Undressed control	Faster early healing with ESM; no long-term difference	By day 21, wounds were fully healed
Ray et al. (2018)	Rat (Wistar)	4 cm^2^ full-thickness skin wound	CS/PCL-modified ESM scaffold	ESM alone; open wound	CP-ESM improved re-epithelialization, collagen and angiogenesis	After 21 days, the CP-ESM group demonstrated successful skin regeneration and 100% wound closure
Saha et al. (2021)	Sprague-Dawley rat	6 mm excision wound	Bilayered gelatin-chitosan-ESM scaffold	Tegaderm; control	Scaffold achieved complete healing by day 14	By day 14, wounds were fully healed in both the bilayered scaffold and Tegaderm groups
Choi et al. (2021)	Sprague-Dawley rat	10 mm full-thickness wound	Acid-modified ESM membrane	Natural ESM; control	Modified ESM accelerated closure and granulation	By day 10, wounds were fully healed
Farman et al. (2021)	Wistar Rat	2 × 2 cm second-degree burn	ESM membrane	Fusidic acid; control	ESM improved epithelialization and burn healing	ESM treatment led to 100% burn wound healing by day 21, superior to fucidin and control
Pillai et al. (2022)	Sprague-Dawley rat	Full-thickness excision	PCL/Chitosan/PVA/SESM dermal patch	Commercial patch	Nanofiber patches healed faster than film	Dermal patch showed complete healing within 14days
Chen et al. (2022)	Sprague-Dawley rat	7 mm full-thickness wound	ESM/CuS/CDs nanofiber membrane	Control; NIR groups	Enhanced fibroblast proliferation and ECM repair	By day 10, skin sections showed strong proliferation in the ESM/CuS/CDs and ESM/CuS/CDs + NIR groups
Zhang et al. (2024)	Sprague-Dawley rat	2 cm circular skin wound	Chitosan-OEM hydrogel	Commercial gel; HCS hydrogel	OEM hydrogel showed superior dermal regeneration	By day 14, the HCS/OEM group showed near-complete healing (0.83% ± 0.63%), outperforming HCS (1.98% ± 0.80%) and the control and CM groups (5.75% ± 1.19% and 6.90% ± 2.78%)
Banu et al. (2024)	Rabbit	2 × 2 cm full-thickness wound	ESM + fibrin glue + BM-MSCs	FG alone; control; FG and ESM; FG and BM-MSCs	Combined ESM+MSCs showed maximal contraction	Complete wound healing by 28 days
Vinayak et al. (2023)	Rabbit	10 mm surgical wound	ESM-BAG mats (Zn/Co doped)	Duoderm; control	Treated groups healed faster with ECM maturation	Treated groups showed a faint line at day 11 that vanished by day 21, while control and commercial dressings retained visible lines at days 11 and 14
Roy et al. (2024)	Diabetic rabbit	12 mm incision wound	ESM-ZnCoBAG mat	Negative/positive controls	ESM-ZnCoBAG gave best wound maturation	By day 21, complete wound closure in test group
Li et al. (2016)	Mouse	8 mm full-thickness wound	Cu-BG/ESM film	ESM; control	5Cu-BG/ESM accelerated epithelialization	Complete wound closure was observed in the test group by day 11
Vuong et al. (2018)	Mouse	6 mm excision wound	ESM powder (PEP)	Untreated control	PEP enhanced keratinocyte proliferation	Wounds were fully closed by day 10
Ahmed et al. (2019)	Mouse	6 mm splinted wound	PEP powder	PBS control	Faster closure and collagen deposition	PEP enhanced collagen deposition with minimal inflammation at day 10
Liu et al. (2017)	Murine	4 mm skin wound	EM/AgNP microfiber	Vaseline; EM; EM/PD	EM/AgNPs promoted re-epithelialization	Wounds were completely closed by day 10

**TABLE 3:** Characteristics of *in vitro* studies of ESM as a wound care material.

Author	Material	Form	*In vitro*	Results
Li et al. (2016)	ESM, Cu^2+^ ions, bioactive glass	Film	Human umbilical vein endothelial cells (HUVECs)	The proliferation of HUVECs on xCu-BG/ESM films increased after 1, 3, and 7 days. At day 1, the xcu-BG/ESM films and pure films showed no significant difference in the proliferation of HUVECs. While, the proliferation level of HUVECs on xcu-BG/ESM film was significantly higher at day 3 and 7 than pure ESM films
Liu et al. (2017)	Silver nanoparticles, eggshell membrane	Microfibers	Fibroblasts	The MTT assay showed no visible cytotoxic effects on fibroblasts, no significant differences in cell viability between EM/PD and EM/AgNPs, and EM/PD exhibited improved cell attachment and vitality when compared to EM.
Vuong et al. (2018)	Eggshell membrane powder (PEP)	Powder	Dermal fibroblast	After 1 day, a notable increase in proliferation at 1 mg/mL was seen without affecting cell viability. Cell proliferation was increased by 1 mg/mL of PEP as early as day 1 and continued to be so at all timepoints (1,3,6 and 10 days). While greater PEP concentrations resulted in decreased viability
Ray et al. (2018)	Chitosan/polycaprolactone (CS/PCL), ESM	Scaffold	Human dermal Fibroblast (HDF) cells	CPESM samples showed higher HDF cell viability than ESM AA samples. After 7 days of incubation, ESM AA and CPESM showed 90% and 140% cell viability respectively. Surface modification with CS/PCL nanofibers significantly enhances scaffold cytocompatibility
Amirsadeghi et al. (2021)	Polyvinyl alcohol, chitosan, SEP, polyethylene oxide, gelatin, zinc oxide nanoparticles	Scaffold	Human fibroblast cells	Fibroblast cell culture on different groups of scaffolds demonstrated that scaffolds have well biocompatibility. SEP and ZnO-NPs have improved scaffolds biocompatibility. The cells adhered well, maintaining their spindle shape, confirming the scaffolds’ biocompatibility
Rønning et al. (2020)	Collagen alone and collagen combined with PEP	Powder	Dermal fibroblasts and skeletal muscle cells	Fibroblast and skeletal muscle cells attached, were viable and able to proliferate for 1 and 2 weeks in both scaffolds
Saha et al. (2021)	Eggshell membrane (ESM) powder, gelatin, chitosan	Scaffold (bilayered structure)	MTT assay using HADF cells	A higher cell density in GCEC indicates that it is a more conducive milieu for fibroblast proliferation. Similarly, gelatin-chitosan film (GCF) increased cell proliferation more than tissue culture plate (TCP).
Choi et al. (2021)	Modification of the natural chicken ESM through weak acid treatment	Eggshell membrane	MTT and CCK-8 assay using HADF cells	Based on the results, cell proliferation of HDF cells was time-dependently increased on the modified ESM than the natural ESM by both MTT and CCK-8 assays
Mensah et al. (2021)	ESM-strip, ESM-A0.5, ESM-E0.9	Strip	SV40 immortalized corneal epithelial cells (ihCEC) and corneal mesenchymal stromal cells (C-MSC)	The attachment and dispersion of C-MSC and iHCE cells on different membrane samples indicates that the inner side (LESM-strip, LESM-A0.5, LESM-E0.9) had a smoother topography or a larger surface area, which resulted in higher cell adhesion than the outer side (OESM-strip, OESM-A0.5, OESM-E0.9)
Sheish et al. (2021)	PVPA–ESM	Nanofibers	PC12 cell	PVPA-ESM wound dressings with 0.5–1 wt% rGO content enhanced PC12 cell viability compared to the wound dressings without rGO nanosheets
Been et al. (2021)	eggshell membrane and agarose (Agr), eggshell	Scaffold	Rabbit cartilage cells (rChons) were isolated from female, New Zealand white rabbits	On day 14, rChon cells in the Arg/ESM scaffold revealed a fibroblasts shape virtually all the seeded cells stayed inside the scaffold
Briggs et al. (2022)	ESM-PNIPAAm; ESM-PNIPAAm (AgNP)	Discs	Human dermal fibroblast (HDFa) and mouse dermal fibroblast (L929) cells	Metabolic activity (MTS assay), LDH release, and Live/Dead staining confirmed good cell attachment, spreading, and high viability after 3 days. Notably, for longer-term viability (>5 days), ESM-PNIPAAm and ESM-PNIPAAm (AgNP) maintained higher and sustained cell viability
Chen et al. (2022)	ESM/CuS NPs, CDs, PVP	Electrospun nanofiber membrnae	Human fibroblasts (HS68)	The composite membrane, prepared in different qualities (0.5, 1, 3, 5 mg), showed high viability at 91.3% when 0.5 mg was used. At 2 mg, it showed acceptable activity at 85.1%, indicating low cytotoxicity and potential for biomedicine applications
Vinayak et al. (2023)	ESM, bioactive glass (BAG), bioactive glass/ion-doped (Zn, Co)	Mat	Human dermal fibroblasts (HDF)	HDFs effectively attached and proliferated on ESM mats, showing well-organized actin filaments and nuclei over 14 days. Cytocompatible and conducive to cellular attachment and spreading
Mensah et al. (2023c)	Drug incorporated PLGA microparticles (MPs) and ESM	Scaffold	*In vitro* Franz diffusion cell eye model	The bandage achieved sustained drug release up to 10 days and was found to be biocompatible and non-toxic in a chorioallantoic membrane (CAM) assay. The bandage has great potential for treating chronic wounds
Mensah et al. (2023b)	AC, AM, SC, SM	Circular discs	HDFs and BJ cells	Modifications of ESM with green-synthesized AgNPs (AM and SM) samples show promising results with lower cytotoxicity profiles than commercial AgNPs. Biological studies showed that the 5 μg/mL AgNPs-ESM samples were highly biocompatible with both HDFs and BJ cells, and had good viability and proliferation rates
Zhang et al. (2024)	Chitosan, oxidized eggshell membrane	Hydrogel	Rat fibroblast cells (L929)	HCS/OEM hydrogel group displayed a considerable increase in cell count on day 5, compared to day 3 and the HCS hydrogel group, suggesting that the OEM addition encouraged cell proliferation
Roy et al. (2024)	ESM-BAG; ESM-ZnBAG; ESM-CoBAG, and ESM-ZnCoBAG mats	Mats	HDF cells	In all mats, including the control (TCP), HDF cell proliferation rose from day 1 to day 14, with the exception of ESM/CoBAG mats, where cell proliferation slightly declined after day 7

Abbreviations: Chitosan/polycaprolactone (CS/ PCL); CS/PCL nanofiber modified bilayered scaffold of extracted air-dried membranes (CPESM); Polycaprolactone/chitosan/polyvinyl alcohol/soluble eggshell membrane protein (PCL/Chitosan/PVA/SESM); Eggshell membrane/Copper sulfide nanoparticles/fluorescence powder/Polyvinylpyrrolidone (ESM/CuS/CDs/PVP); Oxidized eggshell membrane (OEM); Chitosan-based hydrogel (HCS); Bone marrow-derived mesenchymal stem cells (BM-MSCs); Fibrin glue (FG); Bioactive glass (BAG) coated over the eggshell membrane (ESM-BAG); Zinc and cobalt-doped BAG coated over the eggshell membrane (ESM- ZnCoBAG); Copper-Bioactive glass/Eggshell membrane (Cu-BG/ESM); Polydopamine (PD); Processed eggshell membrane powder (PEP); Silver nanoparticles (AgNPs); 3-(4,5-dimethylthiazol-2-yl)-2,5-diphenyltetrazolium bromide (MTT); ESM treated with acetic acid (ESM AA); Human Adrenal Fibroblasts (HADF); Gelatin–chitosan ESM crosslinked (GCEC); Gelatin–chitosan film (GCF); Gelatin–chitosan glutaraldehyde crosslinked (GCGC); Cell Counting Kit-8 (CCK-8); ESM extraction using acetic acid (ESM-A0.5); ESM extracted through ethylenediaminetetraacetic acid (ESM-A0.9); Polyvinylpyrrolidone-acrylic acid (PVPA); reduced graphene oxide (rGO); PNIPAAm, poly(N-isopropylacrylAmide (PNIPAAm); Poly [lactic-co-glycolic acid] (PLGA); Acetic acid extracted ESM incorporated with commercial AgNPs (AC); Acetic acid extracted ESM incorporated with Metalchemy AgNPs (AM); Manually peeled ESM incorporated with commercial AgNPs (SC); Manually peeled ESM incorporated with Metalchemy AgNPs (SM).

Eggshell membrane was applied in multiple formulations, including membrane (Dupoirieux et al., 2001; Guarderas et al., 2016; Choi et al., 2021; Farman et al., 2021; Banu et al., 2024), scaffold (Ray et al., 2018; Saha et al., 2021), dermal patch (Pillai et al., 2022), microfibers (Liu et al., 2017), nanofiber (Chen et al., 2022), hydrogel (Zhang et al., 2024), mat (Vinayak et al., 2023; Roy et al., 2024), film (Li et al., 2016), powder (Vuong et al., 2018; Ahmed et al., 2019) and capsules (Casado-Santos et al., 2024).

*In vitro* experiments were conducted using a wide range of cell lines and models: human umbilical vein endothelial cells (HUVECs) (Li et al., 2016), fibroblasts (Ray et al., 2018; Liu et al., 2017; Vuong et al., 2018; Amirsadeghi et al., 2021), HADF (Choi et al., 2021; Saha et al., 2021; Briggs et al., 2022), L929 cells (Zhang et al., 2024; Briggs et al., 2022), corneal epithelial cells (Mensah et al., 2021), PC 12 cell (Sheish et al., 2021), rabbit cartilage cells (Been et al., 2021), human fibroblasts (HS68) (Chen et al., 2022), HDF (Vinayak et al., 2023; Roy et al., 2024; Mensah et al., 2023b), BJ cells (Mensah et al., 2023b), chondrocytes (Casado-Santos et al., 2024), and an *in vitro* Franz diffusion cell eye model (Mensah et al., 2023c).

## 4 Discussion

**FIGURE 2:**
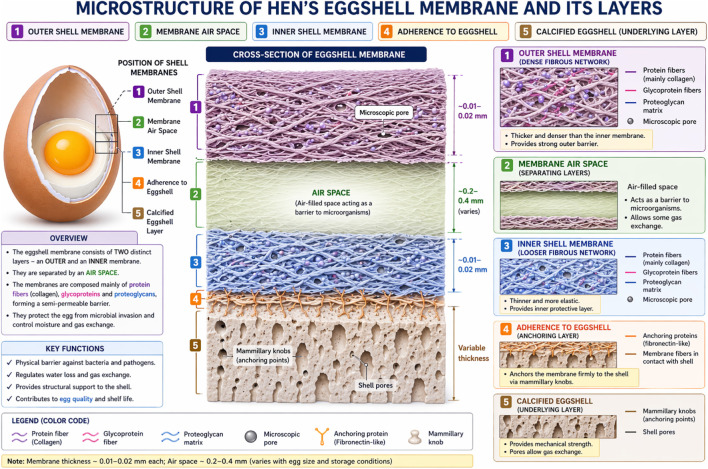
Showing ultrastructure of eggshell membrane.

Recent advances have driven the development of bioengineered skin substitutes based on synthetic polymers, natural biomaterials, or their combinations, offering effective wound coverage while minimizing inflammatory responses. In this context, the ESM is emerging natural biomaterial for wound healing applications. Structurally, ESM is a fibrous, protein-rich tissue located between the mineralized eggshell and the egg white (albumin). It is composed of three distinct layers: the outer shell membrane, inner shell membrane, and limiting membrane (Ahmed et al., 2019; Bellairs and Boyde, 1969). The outer shell membrane, positioned directly beneath the eggshell, contains relatively thick fibers (1–7 µm) that anchor into the shell’s mammillary knobs, while the inner shell membrane is characterized by thinner, finely interwoven fibers (0.1–3 µm) (Ahmed et al., 2019; Zhao and Chi, 2009; Wu et al., 2004) (Figure 2). The innermost limiting membrane forms a dense, non-fibrous layer that encloses the albumen. This hierarchical and organized architecture underpins the mechanical integrity and functional performance of ESM, reinforcing its potential for translational biomedical applications, particularly in the development of bioactive wound dressings. The discussion focused on the mechanism of action and available evidence in classified way to understand how preclinical evidence is derived from included studies.

### 4.1 Mechanism of action of eggshell membrane (ESM) in wound healing

**FIGURE 3:**
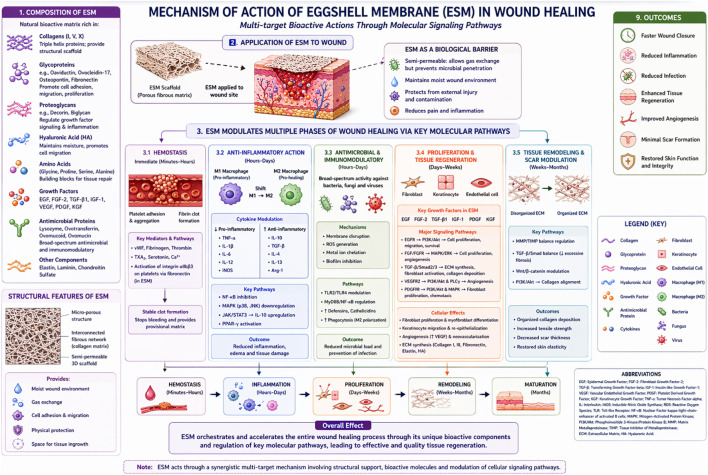
Schematic presentation of mechanism of action of ESM in wound healing.

Eggshell membrane (ESM) is emerging biologically active scaffold with multifactorial regenerative properties. Importantly, a distinction must be made between native ESM and modified/composite ESM systems, as their biological effects may differ significantly. Native ESM primarily functions as a structural extracellular matrix (ECM) analogue, whereas modified forms (e.g., nanoparticle-doped, nanofiber-coated, or hydrogel-integrated ESM) may exhibit enhanced or additional bioactivity. *In vitro* and *in vivo* evidence suggests that ESM is associated with accelerated wound repair through coordinated modulation of cellular proliferation, ECM remodeling, inflammatory responses, and angiogenesis (Figure 3). The mechanistic pathways underlying these effects are discussed below.

#### 4.1.1 Enhanced fibroblast and keratinocyte proliferation

*In vitro* investigations have demonstrated increased proliferation and viability of fibroblasts, keratinocytes, and endothelial cells cultured on ESM-based materials (Li et al., 2016; Vuong et al., 2018; Ray et al., 2018; Zhang et al., 2024). These cellular-level findings are associated with improved *in vivo* healing outcomes, including increased thickness and maturity of granulation tissue, accelerated re-epithelialization, and wound closure within 10–21 days in most animal models.

Enhanced human dermal fibroblast (HDF) proliferation in modified ESM scaffolds was associated with 100% wound closure and histologically observed dermal regeneration by day 21 in rat models (Ray et al., 2018). Similarly, processed eggshell membrane powder (PEP-ESM) that stimulated keratinocyte proliferation *in vitro* correlated with accelerated wound contraction and increased collagen deposition in murine models (Vuong et al., 2018). These findings suggest, but do not establish causality, that early stimulation of dermal and epidermal cell proliferation may contribute to faster wound coverage and granulation matrix formation.

##### 4.1.1.1 Evidence from *in vitro* studies

*In vitro* studies have extensively explored the biocompatibility, cytotoxicity, and cellular responses of ESM based materials, both unmodified and chemically or physically modified, demonstrating their potential for regenerative medicine applications. These investigations have primarily focused on fibroblasts, endothelial cells, and other tissue-specific cell types to assess ESM scaffolds’ capacity to support adhesion, proliferation, and tissue-specific functions.

Li et al. reported that human umbilical vein endothelial cells (HUVECs) cultured on copper-doped bioactive glass-modified ESM (xCu-BG/ESM) exhibited enhanced attachment and active cell morphology after 24 h, as observed by confocal microscopy (Li et al., 2016). Proliferation increased progressively from day 1 to day 7, with 2Cu-BG/ESM and 5Cu-BG/ESM films supporting significantly higher proliferation than pure ESM. Importantly, angiogenesis-related genes [Vascular endothelial growth factor (VEGF); Hypoxia-inducible factor (HIF); VEGF receptor 2 (KDR); Endothelial nitric oxide (eNos)] were upregulated in copper-incorporated films, highlighting the potential of such modifications for vascularization in tissue engineering (Li et al., 2016).

Silver nanoparticle (AgNP) incorporation into ESM also influences cytocompatibility. Liu et al. demonstrated that EM/AgNP composite films exhibited strong cytotoxicity at high silver nitrate concentrations (≥1 mM), whereas lower concentrations (10–100 μM) supported fibroblast attachment, with 30 μM showing optimal cytocompatibility (Liu et al., 2017). MTT assays confirmed comparable fibroblast viability between EM/PD and EM/AgNPs at day 3, emphasizing that nanoparticle concentration critically balances antibacterial activity with biocompatibility (Liu et al., 2017).

The effect of processed ESM powders (PEP) on fibroblast behavior has also been examined. Vuong et al. found that PEP significantly enhanced human dermal fibroblast (HDF) proliferation by day 1 without compromising viability (Vuong et al., 2018). Although cytoskeletal organization and vinculin-positive focal adhesions were altered, matrix remodeling was promoted via increased alpha-smooth muscle actin (α-SMA) and matrix metalloproteinase-2 (MMP-2) activation over 10 days, suggesting the utility of PEP for wound healing applications (Vuong et al., 2018).

Surface modifications of ESM scaffolds further enhance cytocompatibility. Ray et al. showed that HDFs cultured on chitosan/polycaprolactone nanofiber-coated ESM (CP-ESM) exhibited superior adhesion, migration, and proliferation compared to ESM treated with acetic acid (ESM AA) (Ray et al., 2018). CP-ESM scaffolds achieved nearly 140% cell viability by day 7, highlighting the impact of surface modifications on cellular responses. Similarly, Amirsadeghi et al. observed enhanced fibroblast proliferation on ESM scaffolds containing zinc oxide nanoparticles, with preserved cell morphology confirmed by Scanning Electron Microscope (SEM), supporting the biocompatibility of functionalized ESM (Amirsadeghi et al., 2021).

Rønning et al. extended these findings to skeletal muscle cells, demonstrating that both fibroblasts and muscle cells-maintained phenotype markers (TE7, α-SMA for fibroblasts; CD56 for skeletal muscle cells) and showed increased extracellular matrix production on ESM scaffolds (Rønning et al., 2020). Reduced HSP levels further indicated low cellular stress and favorable culture conditions.

The mechanical properties of ESM-based materials also influence cellular behavior. Saha et al. compared gelatin-chitosan films and crosslinked cryogels (GCGC, GCEC) and found that stiffer cryogels improved cell spreading, while GCEC cryogels supported higher cell density (Saha et al., 2021). Although proliferation rates were lower than TCP controls, GCEC provided a more favorable microenvironment for fibroblast growth (Saha et al., 2021).

Chemical modifications of ESM consistently enhance adhesion and proliferation. Choi et al. reported that modified ESM improved hDF cell attachment and proliferation over natural ESM, as confirmed by acridine orange/ethidium bromide (AO/EB) staining and MTT/CCK-8 assays (Choi et al., 2021). Mensah et al. evaluated inner and outer ESM layers and found that the inner side (LESM) supported superior cell adhesion and spreading, likely due to smoother topography, with linear increases in mitochondrial activity over 7 days and no evidence of apoptosis (Mensah et al., 2021).

Incorporation of nanomaterials into ESM can further modulate biocompatibility. Sheish et al. showed that PVPA–ESM nanofibers containing 0.5 wt.% reduced graphene oxide (rGO) achieved cell viabilities >85%, whereas higher concentrations (2 wt.% rGO) reduced viability due to agglomeration (Sheish et al., 2021). These results underscore the importance of optimizing nanomaterial content to promote adhesion and proliferation.

Additional studies confirm the versatility of ESM composites. Been et al. developed agarose/ESM (Agr/ESM) scaffolds that supported 35% higher chondrocyte proliferation by day 14 compared to pure agarose (Been et al., 2021). Briggs et al. (2022) and Chen et al. (2022) also demonstrated that modified ESM and electrospun nanofiber membranes exhibited low cytotoxicity and preserved fibroblast viability, reinforcing their suitability for wound healing. Vinayak et al. (2023) and Roy et al. (2024) reported that bioactive glass and Zn/Co-doped ESM mats promoted continuous fibroblast proliferation, except for cobalt-specific mats, emphasizing the potential of bioactive modifications to enhance regenerative outcomes.

Finally, Mensah et al. validated the biocompatibility and angiogenic potential of ESM samples loaded with VEGF and PLGA microparticles *in vitro* and in ovo (CAM assay), highlighting the capacity of functionalized ESM scaffolds to support vascularization, a critical step in wound healing (Mensah et al., 2023b; Mensah et al., 2023c). Zhang et al. similarly demonstrated that HCS/OEM hydrogels promoted fibroblast proliferation without cytotoxicity, with excellent hemocompatibility and adhesion properties (Zhang et al., 2024).

Collectively, these studies demonstrate that ESM-based scaffolds, particularly when modified through chemical crosslinking, surface functionalization, or nanoparticle incorporation, consistently support cellular attachment, proliferation, and tissue-specific functions. Modifications such as copper doping, nanofiber coatings, and bioactive glass incorporation can significantly enhance angiogenesis, matrix remodeling, and cell viability, underscoring ESM’s potential as a versatile biomaterial for regenerative medicine.

The structural improvements are consistent with *in vitro* observations of enhanced fibroblast metabolic activity, cytocompatibility, and scaffold-guided matrix organization on ESM substrates. By providing a bioactive, collagen-rich matrix environment, ESM may facilitate fibroblast-mediated ECM synthesis and alignment.

The improved ECM maturation directly reduces scar visibility, faster restoration of dermal structure, near-complete epithelial integrity by days 14–21. Molecular-level enhancement of fibroblast function translates into structurally superior tissue remodeling at the macroscopic level, contributing to both functional and aesthetic recovery. However, it is important to note that enhanced ECM outcomes are often more pronounced in modified or composite ESM systems, indicating that bioactive additives (e.g., metal ions, nanofibers) may contribute significantly beyond the intrinsic properties of native ESM.

#### 4.1.2 Upregulation of extracellular matrix production

*In vivo* studies have reported increased deposition of collagen, elastin, and reticulin fibers in ESM-treated wounds (Ahmed et al., 2019; Vinayak et al., 2023; Roy et al., 2024). Histological analysis frequently demonstrates denser and more organized collagen bundles, along with improved dermal architecture compared to controls (Ahmed et al., 2019; Vinayak et al., 2023; Roy et al., 2024).

#### 4.1.3 Modulation of inflammation

Effective wound healing requires a tightly regulated inflammatory response. Excessive or prolonged inflammation delays the transition to the proliferative phase and may impair tissue regeneration. Studies by Liu et al., and Ahmed et al., reported reduced or controlled inflammatory responses in ESM-treated wounds, including modulation of pro-inflammatory cytokines such as interleukin-1β and reduced inflammatory cell infiltration (Liu et al., 2017; Ahmed et al., 2019).

This immunomodulatory effect is associated with resolution of the inflammatory phase and promotes timely progression to tissue regeneration. Clinically and macroscopically, this corresponds to faster wound contraction, reduced erythema and edema, and earlier wound closure. Inflammation regulation provides a biologically plausible bridge between molecular findings (cytokine modulation and reduced inflammatory infiltrate) and visible acceleration of wound healing. Nevertheless, mechanistic insights remain limited, as most studies rely on qualitative or semi-quantitative assessments, and detailed characterization of immune pathways (e.g., macrophage polarization) is lacking. Therefore, causal relationships between ESM and immune modulation remain insufficiently established.

#### 4.1.4 Enhanced angiogenesis

Angiogenesis is critical for delivering oxygen and nutrients to regenerating tissue. *In vivo* cluster of differentiation 31 (CD31) immunostaining and neovascularization assessments demonstrate significantly increased vascular density in modified ESM-treated wounds (Ray et al., 2018). Complementary *in vitro* findings of enhanced endothelial cell proliferation on ESM matrices further support this vascular mechanism (Li et al., 2016). Enhanced vascularization sustains fibroblast activity, collagen synthesis, and keratinocyte migration, ensuring stable re-epithelialization and durable wound closure. ESM accelerates repair by promoting cell proliferation, extracellular matrix deposition, angiogenesis, and controlled inflammation, thereby improving tissue architecture and reducing scarring. These findings suggest a correlation between ESM-based scaffolds and angiogenic activity; however, direct molecular mechanisms remain incompletely defined.

##### 4.1.4.1 *In vivo* evidence from rat models

*In vivo* rat studies demonstrate a progressive refinement of membrane-based strategies for wound healing, evolving from unmodified natural scaffolds to engineered multifunctional systems. Native eggshell membrane (ESM) exhibits baseline regenerative potential, yet its efficacy is modest and context-dependent. Guarderas et al. reported accelerated early wound closure with unmodified ESM, attributed to its fibrous extracellular matrix–like architecture, though healing rates later converged with controls; methodological confounders such as antibiotic use limit interpretation (Guarderas et al., 2016).

Subsequent engineering approaches markedly improved outcomes. Ray et al. showed that bilayered CP–ESM scaffolds enhanced closure, collagen deposition, and neovascularization compared to air-dried ESM, underscoring the importance of nanostructural augmentation (Ray et al., 2018). Chemical modification strategies further advanced bioactivity: acid-treated ESM promoted fibroblast adhesion and angiogenesis without compromising structure (Choi et al., 2021), while Farman et al. demonstrated superior burn healing with native ESM relative to fusidic acid (Farman et al., 2021). These findings highlight both intrinsic antimicrobial properties and the potential of targeted chemical optimization.

Composite scaffold designs expanded functionality. Saha et al.’s gelatin–chitosan cryogel achieved complete closure by day 14 with enhanced angiogenesis, though lacking direct comparison to ESM systems (Saha et al., 2021). Pillai et al. addressed this gap by incorporating soluble ESM proteins into multifunctional nanofibrous patches, achieving superior dermal–epidermal restoration, thereby shifting emphasis from structural to biochemical contributions (Pillai et al., 2022). Chen et al.’s ESM/CuS membranes with carbon dots and NIR irradiation provided photothermal antibacterial activity alongside regenerative support, reducing infection and inflammation in compromised wounds (Chen et al., 2022). Zhang et al. further demonstrated that oxidized ESM within chitosan hydrogels improved adhesion, hemostasis, and organized dermal regeneration, achieving near-complete closure by day 14 (Zhang et al., 2024).

Collectively, these investigations delineate a trajectory from limited efficacy of unmodified ESM to multifunctional hybrid biomaterials with enhanced regenerative capacity. While structural, chemical, and bioactive modifications clearly amplify therapeutic outcomes, variability in experimental design, absence of standardized controls, and limited long-term evaluation constrain translational conclusions. Future work should prioritize direct comparative studies, mechanistic elucidation, and standardized *in vivo* models to define the clinical potential of ESM-based scaffolds.

##### 4.1.4.2 Evidence from *In vivo* studies in mice

Several *in vivo* studies using mouse models have examined the efficacy of biomaterials in wound healing. Li et al. evaluated xCu-BG/ESM and ESM films in a full-thickness skin wound model using female C57/BL6 mice (Li et al., 2016). Among the treatments—ESM, 0Cu-BG/ESM, and 5Cu-BG/ESM—wounds treated with 5Cu-BG/ESM exhibited significantly faster closure and markedly higher vessel density at day 7, indicating enhanced angiogenesis. Histochemical analyses confirmed near-complete epithelialization and a more uniform epidermis in the 5Cu-BG/ESM group, underscoring copper’s pivotal role in neovascularization and wound closure.

Complementary findings were reported by Vuong et al., who investigated PEP in male C57BL/6J mice using a full-thickness wound splinting model (Vuong et al., 2018). PEP significantly increased MMP-2, MMP-9, and MT1-MMP activity at wound edges, highlighting their role in tissue remodeling. Elevated keratinocyte density and accelerated closure further supported PEP’s therapeutic potential, though broader validation across wound types remains necessary (Vuong et al., 2018).

Ahmed et al. reinforced these observations in an excisional wound splinting model with 38 male C57BL/6J mice. PEP treatment accelerated closure, increased granulation tissue thickness at days 10 and 17, and promoted re-epithelialization (Ahmed et al., 2019). KI67 staining confirmed proliferative activity without cytotoxicity, aligning with Vuong et al. and strengthening evidence for PEP’s role in early healing and regeneration (Vuong et al., 2018).

Collectively, these studies highlight the promise of Cu-doped BG/ESM composites and PEP in enhancing angiogenesis, granulation tissue formation, and epithelial regeneration. Nonetheless, further investigations are required to delineate mechanisms, assess long-term outcomes, and validate efficacy across diverse wound models and clinical contexts.

##### 4.1.4.3 Evidence from *In vivo* studies in murine

Liu et al. evaluated EM/AgNPs in a murine full-thickness wound model, revealing accelerated healing relative to controls. While early closure rates were comparable across groups, by day 3 EM/AgNPs achieved significantly greater wound reduction, sustaining superiority through day 7 (88.6% vs. 78.3% in controls, with intermediate outcomes in vaseline gauze, EM, and EM/PD groups) (Liu et al., 2017). Inflammation persisted in non-AgNP groups during the initial 5 days, underscoring the anti-inflammatory advantage of AgNP incorporation. Histology confirmed longer regenerated epidermis and enhanced granulation tissue formation in EM/AgNPs, with EM/PD showing partial benefit (Liu et al., 2017). Immunohistochemistry and Western blotting demonstrated increased proliferating cell nuclear antigen expression, particularly in EM/AgNPs, indicating heightened keratinocyte proliferation at wound margins (Liu et al., 2017).

Collectively, these findings highlight that while EM alone provides structural support, integration with AgNPs confers superior regenerative and anti-inflammatory effects, positioning EM/AgNPs as a more potent scaffold design. Nonetheless, the reliance on short-term murine data necessitates cautious interpretation, and future studies should address durability, mechanistic pathways, and translational relevance.

##### 4.1.4.4 Evidence from *in vivo* studies in rabbit

*In vivo* studies in rabbit models provide compelling evidence for the biocompatibility and regenerative potential of eggshell membrane (ESM)-based biomaterials in wound healing and tissue repair. These investigations consistently demonstrate that ESM functions as an effective scaffold, particularly when combined with bioactive agents or stem cells.

Vinayak et al. evaluated bioactive glass (BAG)-doped ESM mats (ESM/BAG, ESM/ZnBAG, ESM/CoBAG, and ESM/ZnCoBAG) in full-thickness wounds (Vinayak et al., 2023). ESM-treated wounds maintained a moist environment, showed healthy granulation tissue, and achieved complete epithelial closure by day 21, unlike untreated and commercial controls (Vinayak et al., 2023). Histologically, ESM groups exhibited reduced inflammation, enhanced matrix deposition, and complete re-epithelialization (Vinayak et al., 2023).

However, the absence of long-term and mechanistic analyses limits interpretation of sustained regenerative effects. In a diabetic wound model, Roy et al. reported that nanoscale BAG-coated ESM mats accelerated healing, with complete closure by day 21 (Roy et al., 2024). The ZnCo-doped variant showed superior outcomes, including organized collagen deposition, restored dermal architecture, enhanced elastic fibers, and increased glycosaminoglycans and reticulin fibers, indicating improved extracellular matrix remodeling. Nonetheless, the study does not address dose optimization or potential ion-related cytotoxicity.

Banu et al. demonstrated enhanced regeneration using ESM combined with bone marrow-derived mesenchymal stem cells (BM-MSCs). While all groups showed early inflammation, MSC-treated groups exhibited faster epithelialization, reduced inflammation, and increased epithelial thickness. By day 28, the ESM + BM-MSC group showed the greatest improvements in collagen density and tissue organization, supported by Masson’s trichrome staining (Banu et al., 2024). However, the individual contributions of ESM and MSCs and the underlying mechanisms remain unclear.

Overall, ESM-based biomaterials show strong potential for enhancing tissue regeneration, particularly when functionalized or combined with cell therapies. However, variability in scaffold preparation, experimental design, and short follow-up periods limit comparability and clinical translation. Standardized protocols and long-term mechanistic studies are needed to define optimal formulations and validate therapeutic efficacy.

##### 4.1.4.5 Integration of cellular responses with tissue-level outcomes

A central finding across *in vitro* studies is the cytocompatibility of ESM-based scaffolds. These findings are broadly consistent with *in vivo* observations of enhanced granulation tissue formation and re-epithelialization. Modified constructs such as nanofiber-reinforced membranes, ion-doped bioactive glass composites, and oxidized or acid-treated ESM—consistently showed higher metabolic activity and cell viability compared to native membranes.

Importantly, these cellular observations are not isolated phenomena but are reflected *in vivo*. Enhanced fibroblast proliferation observed *in vitro* corresponds with thicker and more mature granulation tissue in animal models. Similarly, increased endothelial cell proliferation aligns with reported improvements in neovascularization, as evidenced by CD31 staining and histological vascular density. These parallels suggest a biologically plausible progression from scaffold-induced cellular activation to tissue regeneration. However, these associations should be interpreted cautiously, as most studies do not establish direct mechanistic causality.

#### 4.1.5 Extracellular matrix deposition and remodeling

A recurring histological outcome across *in vivo* studies is increased collagen deposition, improved collagen organization, and enhanced ECM maturation in ESM-treated wounds. This finding is consistent with *in vitro* evidence of increased fibroblast metabolic activity and scaffold-guided cell alignment. The improved dermal architecture and near-complete epithelial continuity observed by days 10–21 in animal models likely result from this augmented matrix synthesis. Notably, composite ESM systems (e.g., ion-doped or nanofiber-modified scaffolds) frequently demonstrate superior outcomes compared to native ESM, reinforcing the importance of distinguishing intrinsic versus engineered effects.

#### 4.1.6 Modulation of inflammation and early healing dynamics

Controlled inflammatory responses are essential for optimal wound repair. Several studies report reduced inflammatory infiltrate or regulated expression of inflammatory markers in ESM treated wounds. This suggests that ESM may facilitate an earlier transition from the inflammatory to proliferative phase of healing.

Nevertheless, inflammatory assessments are frequently qualitative or semi-quantitative. Few studies measure comprehensive cytokine profiles or characterize immune cell phenotypes. As such, while reduced inflammation correlates with faster closure and improved granulation, the immunomodulatory mechanisms of ESM remain incompletely characterized. However, the lack of comprehensive cytokine profiling and immune cell characterization limits mechanistic interpretation.

#### 4.1.7 Angiogenesis and vascular support

Enhanced neovascularization is frequently reported in the included studies. These findings are particularly evident in modified ESM systems, suggesting that angiogenic effects may be driven in part by incorporated bioactive components rather than ESM alone.

### 4.2 Methodological considerations and risk of bias for *in vitro* and *in vivo* studies

The QUIN risk of bias assessment rated all included *in vitro* studies as having a moderate overall risk (Supplementary Table S2). While study aims, methodology, outcome measurement, statistical analysis, and results reporting were generally low risk, most studies lacked information on sample size calculation, randomization, blinding, and operator/assessor calibration, resulting in high or unclear risk in these domains. These limitations may affect internal validity, despite generally consistent and promising findings.

The *in vivo* studies were judged to have a moderate overall risk of bias (Supplementary Table S3). Although most reports adequately described study aims, methods, outcome measures, and statistical analyses, they often lacked clear details on randomization, blinding, and assessor/operator roles, raising concerns about potential performance and detection bias.

This review examines both *in vitro* and *in vivo* evidence on eggshell membrane (ESM)-based biomaterials for wound healing, critically assessing how molecular and cellular findings translate into macroscopic repair. Collectively, the data demonstrate consistent improvements in wound closure, granulation tissue formation, and extracellular matrix (ECM) remodeling in ESM-treated groups. However, the strength of mechanistic causality varies across studies and requires cautious interpretation.

Despite encouraging outcomes, methodological limitations temper the strength of conclusions. The risk of bias assessments (Supplementary Tables S2, S3) reveal that most *in vitro* and *in vivo* studies demonstrate overall moderate risk of bias primarily due to insufficient reporting of randomization, blinding, and allocation concealment. Blinded outcome assessment is rarely described, and sample size justification is uncommon.

Additionally, heterogeneity in animal models (rats, rabbits, mice), wound types (excision, burn, incision, diabetic models), and scaffold compositions complicates direct comparison. While nearly all studies report improved healing metrics with ESM-based treatments, the variability in endpoints and experimental design limits the ability to perform quantitative synthesis or determine standardized effect sizes.

Another important consideration is translational relevance. Most *in vivo* studies employ acute wound models in otherwise healthy animals. Chronic, ischemic, or infected wound models more representative of clinical reality are underrepresented. Only limited work addresses diabetic conditions. Therefore, while preclinical data are promising, generalizability to chronic human wounds remains to be fully established. Future research should focus on standardized comparative models and metrics to reliably assess clinical translatability, while also exploring long-term outcomes and safety in larger animal models. These limitations may result in overestimation of treatment effects, and therefore, the positive outcomes reported across studies should be interpreted with caution. Additionally, heterogeneity in: animal models, wound types, scaffold compositions limits direct comparability and quantitative synthesis.

### 4.3 Distinguishing structural support from bioactivity

Native ESM exhibits inherent bioactivity due to its collagen-rich composition, while modified or composite forms often demonstrate enhanced regenerative outcomes. Thus, ESM acts as a biocompatible extracellular matrix analogue whose therapeutic potential can be further amplified through functionalization. Recent advances have driven the development of bioengineered skin substitutes based on synthetic polymers, natural biomaterials, or their combinations. In this context, ESM is emerging promising natural biomaterial. Structurally, ESM is a fibrous, protein-rich tissue composed of three layers (Ahmed et al., 2019; Bellairs and Boyde, 1969). While this hierarchical architecture supports its function as a scaffold, variations in extraction and processing methods may alter its structural integrity and biological performance, representing a key source of variability across studies.

## 5 Limitations

Most research is restricted to preclinical models. Variability in ESM processing and lack of standardization remain critical challenges. Importantly, the absence of rigorous experimental design in many studies (e.g., blinding, randomization) reduces confidence in reported outcomes. A major limitation is the predominance of acute wound models in healthy animals. Chronic, ischemic, infected, and diabetic wound models—more representative of clinical scenarios—are underrepresented. Furthermore, direct comparisons with established commercial wound dressings are scarce, limiting assessment of relative clinical efficacy.

## 6 Conclusion

In summary, eggshell membrane represents a promising biomaterial for wound healing applications. Current evidence suggests associations between ESM-based scaffolds and improved wound healing outcomes; however, definitive mechanistic causality and clinical efficacy remain to be established. Preclinical findings provide a foundation for future research, but well-designed studies using standardized models, chronic wound conditions, and clinical comparisons are required to determine translational relevance.

## Data Availability

The raw data supporting the conclusions of this article will be made available by the authors, without undue reservation.
