# Supplementary material for: Eggshell membrane-based biomaterials for tissue regeneration: a systematic review of preclinical evidence
Source: Front Bioeng Biotechnol. 2026 Jun 25;14:1815447. doi: 10.3389/fbioe.2026.1815447 (PMC13346062; doi:10.3389/fbioe.2026.1815447)
Supplement: Supplementary file 1 [file Supplementaryfile1.docx]

Supplementary **Table S1: Excluded Articles with Reasons**

| **S. No.** | **Reference of Article Excluded** | **Reason for Exclusion** |
| --- | --- | --- |
| 1. | Dupoirieux L, Pourquier D, Picot MC, Neves M. Comparative study of three different membranes for guided bone regeneration of rat cranial defects. International Journal of Oral and Maxillofacial Surgery. 2001;30(1):58–62. | Studied only bone healing and regeneration |
| 2. | Durmuş E, Celik I, Ozturk A, Ozkan Y, Aydin M. Evaluation of the potential beneficial effects of Ostrich eggshell combined with eggshell membranes in healing of cranial defects in rabbits. J Int Med Res. 2003 June;31(3):223–30 | Studied only cranial bone defects and their healing |
| 3. | Mogoşanu GD, Grumezescu AM. Natural and synthetic polymers for wounds and burns dressing. International Journal of Pharmaceutics. 2014 Mar 25;463(2):127–36. | Natural and synthetic polymers for wounds |
| 4. | Chen L, Kang J, Sukigara S. Preparation and characterization of polyurethane/soluble eggshell membrane nanofibers. Bio-medical materials and engineering. 2014.24(6):1979–89. | Only mechanical characteristics such as tensile strength and resilience of nanofibers |
| 5. | Li X, Ma M, Ahn DU, Huang X. Preparation and characterization of novel eggshell membrane-chitosan blend films for potential wound-care dressing: From waste to medicinal products. International Journal of Biological Macromolecules. 2019 Feb 15;123:477–84 | Properties such as water resistance, wound fluid absorption, BSA absorption capacity and antibacterial activity that are related to wound healing were improved with the incorporation of 0.01 g/mL ESM and 2% glycerol into chitosan film (2G-0.01ESM/CS). |
| 6. | Li X, Cai Z, Ahn DU, Huang X. Development of an antibacterial nanobiomaterial for wound-care based on the absorption of AgNPs on the eggshell membrane. Colloids and Surfaces B: Biointerfaces. 2019 Nov 1;183:110449. | Only the antibacterial and hydrophilic properties of AgNPs/ESM were observed. Optimized the concentration of AgNPs in the composite. |
| 7. | Ahmed TAE, Suso HP, Hincke MT. Experimental datasets on processed eggshell membrane powder for wound healing. Data Brief. 2019 Aug 31;26:104457. doi: 10.1016/j.dib.2019.104457. PMID: 31667229; PMCID: PMC6811977. | Only the experimental design is provided |
| 8. | Liu S, Huo Z, Zhang H, Hu Q, Ramalingam M. 3D printing-assisted combinatorial approach for designing mechanically-tunable and vascular supportive nanofibrous membranes to repair perforated eardrum. J Appl Polym Sci. 2020;e50132. | Only tested the mechanical properties of ESM |
| 9. | Mendoza N, Chavez G, Araya O. Membrana de cascara de huevo para la curacion de heridas superficiales en ratones. Biomedica. 2022;42:234-43. | The article is not in English language |
| 10. | Webb BC, Rafferty S, Vreugdenhil AJ. Preparation and characterization of antibacterial films with eggshell-membrane biopolymers incorporated with chitosan and plant extracts. Polymers. 2022, 14(3):383 | Only characterization of films is provided |
| 11. | Bello M, Abdullah F, Mahmood WM, Malek NA, Jemon K, Siddiquee S, Chee TY, Sathishkumar P. Electrospun poly (Ɛ-caprolactone)-eggshell membrane nanofibrous mat as a potential wound dressing material. Biochemical Engineering Journal. 2022, 187:108563 | The full-length article is not available |
| 12. | Pawde AM, Sharun K, Shivaramu S, KM M, Paul BR, Dey UK, Maiti SK, Kumar R, Verma MR. Haemato-biochemical alterations associated with the use of eggshell membrane as a dressing material for full-thickness wounds in a rabbit model. Exploratory Animal and medical research. 2023, 13(1). | Hematobiochemical alterations of twenty-five New Zealand White |
| 13. | Casado-Santos A, La Nuez-García MA, Álvarez-Rodríguez P, González-Cubero E, González-Rodríguez Y, Luisa González-Fernández M, et al. Anti-inflammatory and regenerative effects of MKARE® Eggshell Membrane: An *in vitro* osteoarthritis model and placebo-controlled clinical study. Journal of Functional Foods. 2024 May 1;116:106119. | Clinical oral supplementation study in humans for osteoarthritis; not a wound-healing or tissue-repair model. |

Supplementary **Table S2: Risk of Bias Assessment for *in vitro* studies**

| **Study** | **1** | **2** | **3** | **4** | **5** | **6** | **7** | **8** | **9** | **10** | **11** | **12** | **13** |
| --- | --- | --- | --- | --- | --- | --- | --- | --- | --- | --- | --- | --- | --- |
| Li et al., 2016 | Low | High | Unclear | Low | Low | High | High | Low | Unclear | High | Low | Low | **Moderate** |
| Liu et al., 2017 | Low | High | Unclear | Low | Low | High | High | Low | Unclear | High | Low | Low | **Moderate** |
| Vuong et al., 2017 | Low | High | Unclear | Low | Low | High | High | Low | Unclear | High | Low | Low | **Moderate** |
| Ray et al., 2018 | Low | High | Unclear | Low | Low | High | High | Low | High | High | Low | Low | **Moderate** |
| Rønning et al., 2020 | Low | High | Unclear | Low | Low | High | High | Low | Unclear | High | Low | Low | **Moderate** |
| Amir Sadeghi et al., 2021 | Low | High | Unclear | Low | Low | High | High | Low | Unclear | High | Low | Low | **Moderate** |
| Saha et al., 2021 | Low | High | Unclear | Low | Low | High | High | Low | High | High | Low | Low | **Moderate** |
| Choi et al., 2021 | Low | High | Unclear | Low | Low | High | High | Low | High | High | Low | Low | **Moderate** |
| Mensah et al., 2021 | Low | High | Unclear | Low | Low | High | High | Low | Unclear | High | Low | Low | **Moderate** |
| Sheish et al., 2022 | Low | High | Unclear | Low | Low | High | High | Low | Unclear | High | Low | Low | **Moderate** |
| Been et al., 2021 | Low | High | Unclear | Low | Low | High | High | Low | High | High | Low | Low | **Moderate** |
| Briggs et al., 2023 | Low | High | Unclear | Low | Low | High | High | Low | Unclear | High | Low | Low | **Moderate** |
| Chen et al., 2022 | Low | High | Unclear | Low | Low | High | High | Low | High | High | Unclear | Low | **Moderate** |
| Vinayak et al., 2023 | Low | High | Unclear | Low | Low | High | High | Low | Unclear | High | low | Low | **Moderate** |
| Mensah et al., 2023 | Low | High | Unclear | Low | Low | High | High | Low | Unclear | High | Low | Low | **Moderate** |
| Mensah et al., 2023 | Low | High | Unclear | Low | Low | High | High | Low | Unclear | High | Low | Low | **Moderate** |
| Zhang et al., 2024 | Low | High | Unclear | Low | Low | High | High | Low | Unclear | High | Low | Low | **Moderate** |
| Roy et al., 2024 | Low | High | Unclear | Low | Low | High | High | Low | Unclear | High | Unclear | Low | **Moderate** |

Note: 1. Aim 2. Sample size 3. Sampling technique 4. Comparison group 5. Methodology 6. Operator details 7. Randomization 8. Outcome

measurement 9. Assessor Details 10. Blinding 11. Statistics 12. Result presentation 13. Overall ROB

Supplementary **Table S3: Risk of Bias Assessment for *in vivo* studies**

| **Author** | **Selection Bias (Randomization & Allocation)** | **Performance Bias (Housing & Blinding)** | **Detection Bias (Outcome Assessment)** | **Attrition Bias** | **Reporting Bias** | **Overall Risk of Bias** |
| --- | --- | --- | --- | --- | --- | --- |
| Guarderas et al. 2016 | Unclear – randomization and concealment not described | Unclear – no blinding reported | Unclear – assessor blinding not reported | Low | Low | Predominantly unclear |
| Ray et al. 2018 | Unclear – allocation process not described | Unclear – housing and blinding not reported | Unclear – outcome assessor blinding not reported | Low | Low | Predominantly unclear |
| Saha et al. **2**021 | Unclear – no details on random sequence or concealment | Unclear – no blinding or random housing reported | Unclear – outcome assessment not blinded | Low | Low | Predominantly unclear |
| Choi et al. 2021 | Unclear – randomization method not reported | Unclear – caregivers not blinded | Unclear – assessor blinding not stated | Low | Low | Predominantly unclear |
| Farman et al. 2021 | Low – animals stated to be randomly divided; concealment unclear | Unclear – no blinding or random housing | Unclear – assessor blinding not reported | Low | Low | Low to unclear |
| Pillai et al. 2022 | Unclear – randomization and allocation concealment not described | Unclear – no random housing or blinding reported | Unclear – assessor blinding not stated | Low | Low | Predominantly unclear |
| Chen et al. 2023 | Low – explicit random allocation; concealment unclear | Unclear – no blinding or random housing | Unclear – assessor blinding not reported | Low | Unclear | Predominantly unclear |
| Zhang et al. 2024 | Low – explicit random allocation; concealment unclear | Unclear – no blinding reported | Unclear – assessor blinding not stated | Low | Unclear | Predominantly unclear |
| Banu et al. 2023 | Unclear – randomization method not specified | Unclear – no blinding reported | Unclear – assessor blinding not stated | Low | Low | Low to unclear |
| Vinayaka et al. 2023 | Unclear – randomization method not described | Unclear – no blinding reported | Unclear – assessor blinding not mentioned | Low | Low | Predominantly unclear |
| Roy et al. 2024 | Unclear – sequence generation and concealment not described | Unclear – no blinding reported | Unclear – outcome assessor not blinded | Low | Low | Predominantly unclear |
| Li et al. 2016 | Unclear – Randomization method and concealment not described | Unclear – Housing randomization and caregiver blinding not reported | Unclear – Assessor blinding/random order not reported | Low – No exclusions or losses | Low – Methods outcomes reported in Results | Predominantly Unclear |
| Vuong et al. 2018 | Unclear – Random sequence and allocation concealment not described | Unclear – Random housing and blinding not reported | Unclear – No statement on random or blinded outcome assessment | Low – All animals accounted for | Low – Outcomes prespecified and reported | Predominantly Unclear |
| Ahmed et al. 2019 | Unclear – randomization not described | Unclear – no blinding reported | Unclear – assessor blinding not stated | Low | Low | Predominantly unclear |
| Liu et al. 2017 | Unclear – No explicit randomization or allocation concealment | Unclear – Housing and blinding not reported | Unclear – Independent assessors but no blinding stated | Low – n-values consistent at all time points | Low – All stated outcomes reported | Predominantly Unclear |
